# Clinical use of cardiovascular magnetic resonance-defined synthetic extracellular volume fraction

**DOI:** 10.1016/j.jocmr.2025.101891

**Published:** 2025-04-09

**Authors:** David A. Bluemke, Prashant Nagpal

**Affiliations:** Department of Radiology, The University of Wisconsin-Madison, 600 Highland Avenue, Madison, WI 53792, USA

It is quite remarkable that a parameter as physiologically interesting as extracellular volume fraction (ECV) can be easily obtained by cardiovascular magnetic resonance (CMR). As reviewed by Mewton et al., cardiac tissue consists of cardiac myocytes plus the space outside those cells—the extracellular space [1]. In the normal heart, the extracellular space contains the support system of the heart—dominated by capillaries, nerves, lymphatics, and other scattered and less common cell types. In rough terms, approximately 25% of the myocardial mass is extracellular, and 75% cellular. Conventional gadolinium contrast agents distribute only to the extracellular space.

When the heart is injured, the ratio of extracellular and intracellular components changes dramatically. The most common example of this is acute myocardial injury, e.g., due to infarction or inflammation. In both circumstances, troponin regulatory proteins in the bloodstream are elevated because the cell membranes of myocytes have been damaged or destroyed. Massive cell damage from myocardial infarction can result in ECVs as high as 70–80%. In acute myocardial infarction, the extracellular volume is greatly increased by edema and plasma. In chronic healed infarction, large bands of fibrosis develop and replace damaged myocardium [2]. *Replacement fibrosis* is identified by late gadolinium enhancement magnetic resonance imaging as myocardial scar.

Chronic types of myocardial diseases are less dramatic, but can have a similar, devastating impact on myocardial function. Many types of chronic myocardial disease will result in *reactive* fibrosis with resulting diffuse collagen deposition in the absence of myocardial damage. For example, aortic stenosis results in increased wall stress in the left ventricle. Unlike a balloon, which stretches and dilates in response to greater pressure, the myocardium can compensate and resist the tendency to dilate. Under stress, activated fibroblasts secrete triple-stranded procollagen. Outside the cell, collagen peptidases act on procollagen to become tropocollagen. Lysyl oxidase promotes the formation of covalent bonds between tropocollagen molecules, which are then termed collagen fibrils. These fibrils reinforce the heart wall, helping it resist expanding like a balloon—at least for a while. The resulting *reactive interstitial fibrosis* is a common reason for mild elevation of the ECV, e.g., to 30–35%, depending on its severity and duration. Because reactive interstitial fibrosis occurs diffusely throughout the myocardium, it is not identified on late gadolinium CMR images.

*Infiltrative interstitial fibrosis* results from the deposition of insoluble proteins. The most common forms encountered by the CMR physician include amyloid protein deposition (amyloidosis) and glycosphingolipids (Anderson-Fabry disease). In amyloidosis, the extracellular space may be markedly expanded by deposits of amyloid protein. ECV levels of 40–60% may be seen in amyloidosis [3], substantially higher than seen with reactive interstitial fibrosis. Extreme deposits of amyloid protein are identified by late gadolinium CMR, but early disease is not identified.

CMR is now a routine test to estimate myocardial ECV, a fundamental property of tissue health. In a meta-analysis, CMR assessed that ECV was similar (25.9%) at both 1.5T and 3.0T [4]. Major technical factors shown to affect the CMR estimate of ECV were the pulse sequence scheme and the type of gadolinium contrast agent.

Detailed descriptions of the calculation of ECV using CMR have been provided [5], [6]. In brief, we need to measure five parameters. The first four parameters are the relaxivity (R1) of the blood pool and the myocardium both before and after administration of an extracellular gadolinium contrast (note that R1 = 1/T1 relaxation time). Jerosch-Herold et al. showed that there is rapid distribution of standard extracellular contrast agents, so the post gadolinium measurements can be done any time after approximately 4 min or more post-injection [6]. These measurements yield the partition coefficient for gadolinium λ_gad_ = ΔR1 (*myocardium*)/ΔR1 (*blood*), where ΔR1 is the relaxivity post gadolinium minus relaxivity before gadolinium administration.

The fifth parameter needed to calculate ECV is blood hematocrit. The ECV is calculated as ECV = λ_gad_ × (1 − hematocrit (%)/100). A sticking point for clinical practice is the requirement to obtain the patient’s hematocrit level near the time of the CMR study. The hematocrit varies throughout the day. As a result, the Society for Cardiovascular Magnetic Resonance recommends measuring hematocrit immediately before the CMR examination [7]. Many clinical practices find that yet another blood test at the time the CMR is simply not practical to routinely obtain before the CMR examination.

When the hematocrit is not available, it is possible to estimate hematocrit based on the linear relationship of the R1 of blood and hematocrit. That is, if the R1 value of blood is known, the hematocrit can be derived based on a linear regression equation. This approach is called the “synthetic ECV.” The advantages of using synthetic ECV are that ventricular blood is used for both hematocrit estimation and as part of the partition coefficient calculation; the estimate of hematocrit is done at the time of the CMR. This avoids the complication of diurnal and day-to-day variations in hematocrit measured by conventional blood sampling.

In their recent study in *the Journal*, Yasuda et al. [8] performed a meta-analysis of the literature to determine the agreement between synthetic ECV and laboratory-blood sample determined ECV. The authors identified 10 studies (4492 patients) in which both synthetic ECV and laboratory hematocrit ECV values were determined. The correlation between synthetic ECV obtained at 1.5T and laboratory ECV was remarkably high (r = 0.95 [0.92–0.97]). At 3.0T, the correlation was only slightly lower (r = 0.91 [0.86–0.94]). Measures of heterogeneity between published studies were acceptable, indicating the validity of the meta-analysis. The authors evaluated ECV results for healthy controls, cardiac amyloidosis, and other cardiovascular diseases. No substantial differences in synthetic ECV versus laboratory ECV at 1.5T and 3T were found.

Overall, the results of Yasuda et al. are extremely reassuring and support the use of synthetic ECV. Shall we go ahead and immediately implement synthetic ECV as our clinical standard? This would avoid the expense and inconvenience of additional blood sampling before CMR. The answer is both “yes” and “no.” Understanding “yes” is easy: in some cases, synthetic ECV is all that may be available. In that case, synthetic ECV may aid the interpretation of the CMR. Unsuspected amyloidosis may be identified in this manner.

What is the hesitation in using synthetic ECV? As mentioned above, synthetic ECV depends on knowing the regression equation between blood R1 and hematocrit. Although linear, patients who are critically ill or with abnormal blood composition (e.g., excess iron, anemia) will deviate from a linear curve, resulting in incorrect ECV estimates. In addition, the exact linear regression equation between R1 and hematocrit will differ between your CMR scanner and ours. Formulas derived from different scanners and different pulse sequences give different results. For example, Reiter et al. found that application of published formulas for synthetic ECV for different 3T scanners led to incorrect classification in up to 60% of patients [9]. Site-specific formulas to derive hematocrit are thus necessary.

In conclusion, Yasuda et al. provide further evidence to support the use of synthetic ECV in routine practice. Synthetic ECV has a remarkably high correlation with laboratory hematocrit-derived ECV. Synthetic ECV avoids blood sampling and additional costs. Calculations of synthetic ECV are typically built into our existing CMR analysis software packages. However, there are caveats. First, each site must derive its own calibration curve between hematocrit and R1 relaxivity. While somewhat time-consuming, this is less onerous than determining site-specific normal T1 and T2 values (which requires scanning 40–50 healthy volunteers). By comparison, obtaining 40–50 hematocrit values before CMR is straightforward. This calibration needs to be done for each CMR scanner being used. After deriving the calibration curve, pulse sequences and type of gadolinium contrast cannot be changed. CMR physicians also need to be aware that synthetic ECV is likely to be erroneous in patients with severe anemia or iron overload, or in patients with disorders resulting in unusual blood chemistry.

## Funding

Prashant Nagpal: Grant support from 10.13039/100015650Canon Medical.

## Author contributions

**Prashant Nagpal:** Writing – review & editing.

## Declaration of competing interests

The authors declare the following financial interests/personal relationships which may be considered as potential competing interests: David A. Bluemke reports a relationship with GE Healthcare that includes consulting or advisory. David A. Bluemke reports a relationship with Edgewise Therapeutics, Inc. that includes consulting or advisory. Prashant Nagpal reports a relationship with GE Healthcare that includes consulting or advisory. Prashant Nagpal reports a relationship with WCG Clinical that includes consulting. Prashant Nagpal reports a relationship with Moderna that includes stock ownership.
